# Single-Leg Assessment of Postural Stability After Anterior Cruciate Ligament Injury: a Systematic Review and Meta-Analysis

**DOI:** 10.1186/s40798-017-0100-5

**Published:** 2017-08-29

**Authors:** Tim Lehmann, Linda Paschen, Jochen Baumeister

**Affiliations:** 1 Exercise Neuroscience & Health Lab, Institute of Health, Nutrition and Sport Sciences, University of Flensburg, Campusallee 2, 24943 Flensburg, Germany; 2 Exercise Science, Department of Exercise & Health, Faculty of Science, Paderborn University, Paderborn, Germany

## Abstract

**Background:**

Previous reports of single-leg assessment demonstrated functional deficits in postural stability following anterior cruciate ligament (ACL) injury. However, quantified measures describing postural stability vary among investigations and results seem not to be clear. The first aim of this systematic review was to quantify postural deficits in eyes open single-leg stance in patients after ACL injury. Moreover, the second aim was to examine the potential of traditional center of pressure (CoP) measures in order to distinguish postural stability between ACL patients and healthy controls.

**Methods:**

A systematic literature search in the databases PubMed and Scopus was conducted from their inception to December 2016 to identify relevant articles. Eligibility criteria were limited to controlled trials of eyes open static single-leg stance on a force or pressure plate recording CoP measures in patients after ACL injury.

**Results:**

Eleven studies were included, involving a total of 329 ACL-injured and 265 control subjects. Random-effects meta-analysis showed significantly increased sway magnitudes (SMD_wm_ = 0.94, *p =* 0.003) and velocities (SMD_wm_ = 0.66, *p =* 0.0002) in the ACL group compared to the healthy controls. Sway magnitude in anteroposterior (SMD_wm_ = 0.58, *p =* 0.02) and mediolateral (SMD_wm_ = 1.15, *p =* 0.02) direction were significantly increased in ACL patients. No differences were found for the non-injured side. Similarly, no differences have been observed among ACL patients between the injured and non-injured side for sway velocity, while sway magnitude significantly differed (SMD_wm_ = 0.58, *p =* 0.05).

**Conclusions:**

The findings of this systematic review and meta-analysis demonstrated decreased postural stability in individuals with ACL injury. Sway magnitude and velocity were significantly increased in the ACL group compared to the healthy controls. Although the included research still exhibited considerable heterogeneity, it may be proposed that fundamental CoP measures are suitable to differentiate patients after ACL injury and healthy controls with respect to postural stability in eyes open single-leg stance.

## Key Points

- This is the first systematic review and meta-analysis examining gold standard center of pressure measures in patients with injury to the anterior cruciate ligament, irrespective of clinical treatment.
- Injury to the anterior cruciate ligament is associated with increased sway magnitudes and velocities during a standardized single-leg stance in the injured leg.
- Gold standard center of pressure measures allow for a functional distinction between subjects with and without injury to anterior cruciate ligament in terms of postural stability.

## Background

Injuries to the anterior cruciate ligament (ACL) are the most frequent knee injuries in sports and cause immediate disability for athletes followed by long-term consequences in terms of functional deficits in motor coordination [3, 17, 25, 55]. Generally, functional stability of the knee joint during voluntary movement is predominantly regulated by the sensorimotor system. As a dynamic system, it contributes to the transmission and integration of somatosensory, vestibular, and visual information to the central nervous system, in order to provide adaptability to the environment [8, 31, 56]. Alterations of afferent sensory information, potentially caused by mechanoreceptor damage, may subsequently contribute to disturbances of sensorimotor control [1, 25, 59]. Based on this rationale, research has shown altered sensorimotor control after ACL injury [4–6, 10, 15, 19, 44], whereas contradictory findings indicate that these alterations may not necessarily be correlated with postural control of standing balance [15, 23, 34].

Postural control is defined as the ability to monitor body position and alignment in space, involving multimodal interactions of musculoskeletal and neural systems [54]. It is comprised of two components: postural orientation and postural stability. While postural orientation describes the visually and vestibular-guided ability of monitoring the interrelationship between body segments relative to the environment, postural stability predominantly incorporates somatosensory information to control the center of mass (CoM) in relationship to the base of support [54]. To date, center of pressure (CoP) trajectories, as the vector of total force applied to the center of the supporting surface [9, 60], are measured by laboratory-based force or pressure-sensitive platforms in order to assess postural stability [12, 30, 47, 48]. After ACL injury, measures describing postural stability vary among investigations and results seem not to be clear [28].

Postural stability as a crucial determinant for functional movement reflects a multimodal interaction of the sensorimotor system [54]; however, there is no gold standard to assess postural stability in patients after ACL injury. Therefore, the purpose of the present systematic review and meta-analysis was to investigate postural stability after ACL injury. The second aim was to examine the potential of CoP measures to distinguish postural stability between ACL patients and healthy controls. Given that there is no clear consensus about the feasibility of these measures in ACL research, this meta-analysis on postural stability measures may further the development of valid tools to examine functional outcomes after rehabilitation or reconstruction in ACL patients.

## Methods

Conducting this meta-analysis, the Preferred Reporting Items for Systematic Reviews and Meta-Analyses (PRISMA) provided by Moher et al. [39] were followed and adapted to the current data properties.

A systematic literature search in the databases PubMed and Scopus was conducted from their inception to December 2016 to capture all pertinent articles investigating postural stability in ACL patients. The search strategy included the key terms: (postural control OR postural balance OR vestibular OR posture OR balance) AND (“ACL” OR “anterior cruciate ligament”). Since there is no universal definition of postural control and balance, this search strategy comprised a widespread spectrum in order to cover all potentially relevant studies. Search limitations were imposed to full access articles in English language and studies investigating human species. Additionally, reference lists of articles found were inspected, and relevant review articles [20, 28, 41] were scrutinized to identify further evidence.

### Inclusion Criteria

The inclusion criteria for this meta-analysis were as follows: (1) controlled trials of post-injury postural stability in patients after ACL injury, (2) static postural stability tests in single-leg stance utilizing force or pressure plates, (3) subjects of all ages and sexes without any neurological or psychological diseases or history of lower limb musculoskeletal surgery, and (4) investigations reporting at least one primary outcome measure of static postural stability based on the CoP. Due to standardization demands, the testing protocol was limited to ordinary joint loading tasks that allow for functional assessment of the ACL-injured and ACL-non-injured limbs. Therefore, any papers not meeting these criteria, solely investigating dynamic tasks, double leg stance or eyes closed, just as effect or interventional studies were not eligible for inclusion.

### Study Selection

Based on the predetermined inclusion criteria, records were identified and screened through database searching. Records of both databases were then merged, and duplicates were removed using Mendeley Desktop (v.1.17, Mendeley Ltd., London, UK). Two independent reviewers (TL and LP) conducted the study selection. If the included studies did not report means, standard deviations, or *F* values, the corresponding authors were contacted. In two of three cases, the authors responded [18, 57] and the respective study was included, while the remainder [21] was excluded from this meta-analysis.

To assess methodological quality of the studies, a modified Quality Assessment Tool for Observational Cohort and Cross-Sectional Studies [58] was independently applied to each included article in order to assess the internal validity and risk of selection-, information-, or measurement bias. The tool is composed of 14 criteria inspecting the objectives, population, participation, exposures, and outcomes of the particular investigations. In case of this meta-analysis, three criteria (3, 10, 13) were not applicable in relation to the research objectives pursued and therefore excepted from the assessment. The remaining 11 criteria were evaluated on the scale “yes,” “no,” “not applicable,” “not reported,” or “cannot determine,” with any response other than “yes” posing a certain risk of bias. A total score was generated counting all “yes” responses for each study. On the basis of recommendations provided by Aderem and Louw [2], total scores below 50% were considered as “poor,” total scores between 50 and 75% as “fair,” and total scores above 75% as “good” methodological quality. Additionally, funnel plots of the effect size and the standard error were generated for the included trials in order to assess publication bias.

### Outcome Measures

The outcome measures considered in this review correspond to basic descriptions of CoP trajectories with regard to magnitude, direction, and velocity of the displacement [45]:

1. The sway amplitude is the mean of all data points collected for one or more trials.
2. Path line length further represents the total distance traveled by the CoP over the course of a trial.
3. Area of sway describes the total area covered by the CoP in both anteroposterior (AP) and mediolateral (ML) direction.
4. CoP mean velocity is determined as the total distance traveled by the CoP divided by time.
5. The maximum speed is calculated as the peak velocity reached by CoP dislocation across trials.

### Data Extraction

For each study meeting the inclusion criteria, descriptive information related to the country of origin, subject characteristics, sample size, time from injury/surgery to testing, the research protocol, and associated injuries were summarized using a customized Excel (Microsoft, Redmond Washington, USA) spreadsheet. Measures for these data were means and standard deviations. The primary outcome measures for the present meta-analysis were categorized to sway magnitude (sway amplitude, sway area, path length) and sway velocity (mean velocity, maximum speed) in total, AP, and ML direction. In two cases, the data for classified groups of functional recovery [57], just as males and females [53], were matched together by means, since no differentiation was intended for these subgroups of patients in the current meta-analysis. If repeated measures or different conditions were reported, the first or baseline measurement was considered exclusively.

### Statistical Analysis

The main statistical analyses were executed for leg (injured, non-injured, matched) and direction (total, AP, ML) for the parameters sway magnitude and sway velocity. Based on sample size, means/*F* values, and standard deviation, the particular effect sizes were calculated as the standardized mean difference (SMD) for each CoP measure and study [36] in order to examine statistical differences between patients after ACL injury and healthy controls. The SMDs of all studies were then weighted with respect to the magnitude of their standard error (SMD_wm_). Positive effect sizes indicate better postural stability in the control group or leg, while negative effect sizes favor the ACL group.

Using meta-analyses, eight hypotheses were tested for differences in ACL and healthy subjects with regard to sway magnitude, sway velocity, injured vs. matched leg, non-injured vs. matched leg, injured vs. non-injured leg, anteroposterior sway, and mediolateral sway. All comparisons were computed with a random-effects model using Review Manager (v.5.3.5, The Nordic Cochrane Centre, The Cochrane Collaboration, Copenhagen, DK) to calculate the overall standardized mean difference of the respective outcome measures. Further, the 95% confidence interval (CI) was computed for the individual and overall effect. Based on recommendations provided by Cohen [14], effect sizes were interpreted as follows: 0.00 to 0.49 indicate a small effect, 0.50 to 0.79 were considered a medium, and values greater than 0.80 indicate a large effect. Heterogeneity between trials was tested and interpreted using *I* ^2^ percentages. Hereof, the impact of potential heterogeneity on the results of the meta-analysis was estimated referring to suggestions from Higgins [26]: *I* ^2^ values from 30 to 50% indicate moderate heterogeneity, values greater than 50% display a substantial heterogeneity, and values of greater than 75% may be interpreted as considerable heterogeneity.

## Results

### Study Characteristics

**Fig. 1:**
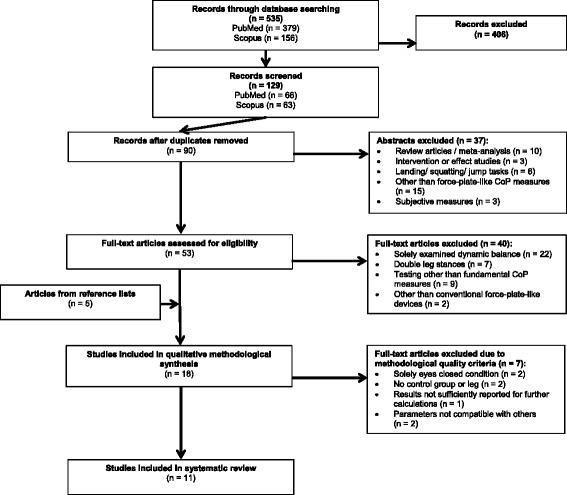
PRISMA flow diagram of literature search process. *CoP* center of pressure

The literature screening revealed a total of 535 records through database searching (Fig. 1). Additionally, five articles [27, 37, 42, 52, 61] were identified from the reference lists of eligible articles. Two of them [27, 52] qualified for inclusion. Ultimately, 11 studies published from 1996 to 2016 were included in the meta-analysis.

These investigations enrolled 594 subjects: 329 patients after ACL injury and 265 healthy controls (Table 1). Mean sample sizes were 29.90 ± 20.03 for the patient group and 24.09 ± 10.38 for the controls. The ACL group comprised 19.89 ± 7.47 males and 10.44 ± 9.54 females on average (ratio 66%/34%). Similarly, the control group was composed of 19.50 ± 8.05 males and 8.00 ± 5.13 females (ratio 70%/30%). Both the ACL and control subjects were physically active for 1–3 days per week [13] or were involved in team sports like soccer and basketball [33, 38, 40, 62].

**Table 1:** Study characteristics of included articles investigating postural stability in single-leg stance

Study	Country	Group	Sample size	Sex	Age, years (mean ± SD/range)	Time from: 1) Injury-test 2) Injury-surgery 3) Surgery-test (mean ± SD/median [range])	Associated injuries	Balance task	Measures/device considered
Clark et al. [13]	Australia	ACLR, ST R/L: 19/24 PRP	45	30 male 15 female	26.0 ± 9.8 (15–53 years)	2) 3.8 ± 3.77 months (0.32–23.97 months) 3) 10.72 ± 4.25 months (5.65–20.4 months)	Meniscus repair (*n* = 10)	SLS, flexion, EO 3 × 30 s	Sway amplitude mean velocity Wii Balance Board
		Healthy matched controls	45	30 male 15 female	26.4 ± 9.8 (15–52 years)		N		
Dauty et al. [16]	France	ACLR, PT/ST R/L: 21/14 PRP	35	26 male 9 female	25.5 ± 5.8	1) 7.4 months (1–13 months)	NR	SLS, flexion, EO 3 × 12 s	Sway area AP path length ML path length Medicapteurs QFP stabilometric platform
		Healthy controls	35	26 male 9 female	24.9 ± 5.9		N		
Fernandes et al. [18]	Brazil	ACLD, CT 14 DL PRP	14	11 male 3 female	25.0 ± 7.2	1) 9.29 ± 9.59 months (3–18 months)	N	SLS, EO 3 × 30 s	Sway area AP amplitude ML amplitude Sway velocity Loran engineering EPS pressure platform
		Healthy controls	14	10 male 4 female	25.0 ± 2.8		N		
Hoffman et al. [27]	USA	ACLR, PT	20	8 male 12 female	23.4 ± 5.79	2) 9.52 months (3–30 months)	CD	SLS, EO 4 × 20 s	Sway path linear mean Kistler force plate
		Healthy matched controls	20	13 male 7 female	24.0 ± 4.07		CD		
Kouvelioti et al. [33]	Greece	ACLR, ST PRP	15	NR	24.37 ± 3.50	3) 24 months	NR	SLS, front raise, EO 1 × 30 s	Sway area AMTI AccuSway force platform
		Healthy controls	10	NR	26.75 ± 2.37		N		
Mohammadi et al. [38]	Iran	ACLR, PT/ST DL/nDL: 16/12 PRP	30	22 male 8 female	25.0 ± 2.7	3) 8.4 ± 1.8 months (6–10 months)	Meniscus injury (*n* = 16)	SLS, flexion, EO 3 × 30 s	AP sway amplitude ML sway amplitude AP sway velocity ML sway velocity mean velocity Kistler force plate
		Healthy matched controls	30	24 male 6 female	24.8 ± 2.4		N		
Negahban et al. [40]	Iran	ACLD, CT	27	27 male	26.74 ± 5.84	1) 21.6 ± 26.76 months	NR	SLS, semiflexion, EO 3 × 20 s	Mean velocity Bertec force platform
		Healthy matched controls	27	27 male	26.29 ± 5.07		N		
Sayuri Tookuni et al. [52]	Brazil	ACLD, CT R/L: 8 ⁄ 11 PRP	19	18 male 1 female	15–33	NR	NR	SLS, flexion, EO 3 × 10 s	Total path length AP path length ML path length maximum speed Tekscan F-Scan mat
		Healthy controls	19	11 male, 8 female	18–30		N		
Shiraishi et al. [53]	Japan	ACLR, IT PRP ACLD, CT	53 30	22 male 31 female 15 male 15 female	25.3 ± 6.6 (18–44) 23.7 ± 5.3 (18–39)	2) 25.2 ± 21.6 months (0.25–84 months) 3) 45.6 ± 18 months (24–72 months) 1) > 4 months	Meniscus injury (*n* = 33)	SLS front raise, EO 3 × 20 s	Total path length ANIMA GS-10 Gravicorder
		Healthy controls	30	15 male 15 female	22.7 ± 2.9 (19–29)		N		
Soltani et al. [57]	Iran	ACLD copper CT ACLD non-copper CT	5 10	NR	26.0 ± 3.0 24.0 ± 2.0	1) 18.2 ± 5.0 months 1) 13.0 ± 7.4 months	N	SLS, EO 5 × 10 s	Path line length Sway velocity Zebris platform
		Healthy matched controls	15	NR	23.7 ± 2.4		N		
Zouita Ben Moussa et al. [62]	Tunisia	ACLR, PT	26	NR	22.0 ± 3.11	3) 24 ± 0.25 months	NR	SLS, EO 3 × 10 s	Sway velocity NeuroCom Balance Master platform
		Healthy matched controls	20	NR	23.96 ± 2.02		N		

*NR* not reported, *N* no, *CD* cannot determine, *ACLD* anterior cruciate ligament deficient, *ACLR* anterior cruciate ligament reconstructed, *CT* conservative treatment, *PT* patellar tendon allograft/autograft, *ST* semitendinosus tendon allograft/autograft, *IT* iliotibial tract autograft, *DL/nDL* dominant leg/non-dominant leg injury, *R/L* right/left sided injury, *PRP* patients completed postsurgical rehabilitation program, *SLS* single-leg stance, *EO* eyes open, *AP* anteroposterior, *ML* mediolateral

According to the type of treatment after injury, 224 patients underwent surgery and 105 patients were treated conservatively. Three studies [13, 27, 53] reported a mean time from injury to surgery of 44.74 ± 54.47 weeks, while the range for all studies varied from 7 days to 7 years. No study outlined muscular or neurological damages, but three studies [13, 38, 53] depicted a total of 59 patients (26.3%) with associated meniscal tears. None of the patients in either group had a history of previous ACL injury. The trial length varied among studies from 10 to 30 s (20.28 ± 9.14) and was measured in one to five sets (2.8 ± 1.08). Total recording time, summing all trials per study, ranged from 30 to 90 s (50.84 ± 23.87).

The quality assessment tool (Table 2) has shown an overall methodological appraisal score of 58%, indicating fair methodological quality according to the predetermined classification criteria. Most studies (9/11) reached a level of fair quality, whereas two studies were classified as poor quality. Furthermore, no evidence for publication bias was found through funnel plot inspection.

**Table 2:** Quality rating based on recommendations of Aderem and Louw [2]

Quality criteria	Clark et al. [13]	Dauty et al. [16]	Fernandes et al. [18]	Hoffman et al. [27]	Kouvelioti et al. [33]	Mohammadi et al. [38]	Negahban et al. [40]	Sayuri Tookuni et al. [52]	Shiraishi et al. [53]	Soltani et al. [57]	Zouita Ben Moussa et al. [62]
1. Was the research question or objective in this paper clearly stated?	Y	Y	Y	Y	Y	Y	Y	Y	N	Y	Y
2. Was the study population clearly specified and defined?	Y	Y	Y	Y	Y	Y	Y	Y	Y	Y	Y
4. Were all the subjects selected or recruited from the same or similar populations (including the same time period)? Were inclusion and exclusion criteria for being in the study prespecified and applied uniformly to all participants?	Y	Y	Y	Y	CD	CD	Y	CD	CD	Y	Y
5. Was a sample size justification, power description, or variance and effect estimates provided?	Y	N	N	N	N	Y	N	N	N	Y	N
6. For the analyses in this paper, were the exposure(s) of interest measured prior to the outcome(s) being measured?	Y	Y	Y	Y	Y	Y	Y	Y	Y	Y	Y
7. Was the timeframe sufficient (range < 24 months) so that one could reasonably expect to see an association between exposure and outcome if it existed?	Y	Y	Y	CD	N	Y	N	NR	N	N	N
8. For exposures that can vary in amount or level, did the study examine different levels of the exposure as related to the outcome (e.g., categories of exposure, or exposure measured as continuous variable)?	NA	NA	NA	NA	NA	NA	NA	NA	Y	Y	NA
9. Were the exposure measures (independent variables) clearly defined, valid, reliable, and implemented consistently across all study participants?	Y	Y	Y	Y	Y	Y	Y	CD	Y	Y	Y
11. Were the outcome measures (dependent variables) clearly defined, valid, reliable, and implemented consistently across all study participants?	Y	Y	Y	Y	Y	Y	Y	Y	Y	Y	Y
12. Were the outcome assessors blinded to the exposure status of participants?	NR	NR	NR	NR	NR	NR	NR	NR	NR	NR	NR
14. Were key potential confounding variables measured and adjusted statistically for their impact on the relationship between exposure(s) and outcome(s)?	N	N	N	N	N	Y	N	N	Y	N	N
Total score	8/11	7/11	7/11	6/11	5/11	8/11	6/11	4/11	6/11	8/11	6/11
Total score (%)	72%	63%	63%	54%	45%	72%	54%	36%	54%	72%	54%
Quality rating [2]	Fair	Fair	Fair	Fair	Poor	Fair	Fair	Poor	Fair	Fair	Fair

*Y* yes, *N* no, *CD* cannot determine, *NR* not reported, *NA* not applicable

### Quantitative Data Synthesis

**Fig. 2:**
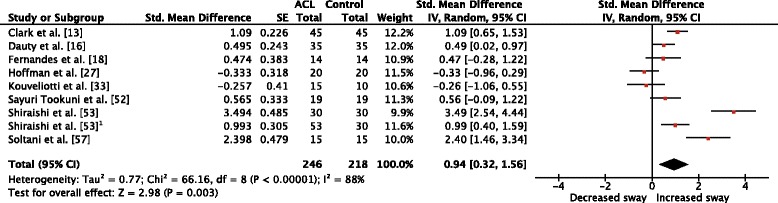
Forest plot comparison of sway magnitude between the ACL-injured and ACL-matched control leg. *Std*. standardized, *SE* standard error, *IV* inverse variance, *CI* confidence interval, *df* degrees of freedom, *ACL* anterior cruciate ligament. ^1^Second ACL group in the same study

**Fig. 3:**
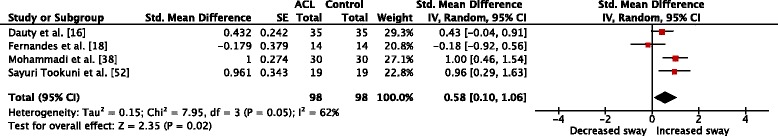
Forest plot comparison of anteroposterior sway magnitude between the ACL-injured and ACL-matched control leg. *Std*. standardized, *SE* standard error, *IV* inverse variance, *CI* confidence interval, *df* degrees of freedom, *ACL* anterior cruciate ligament

**Fig. 4:**
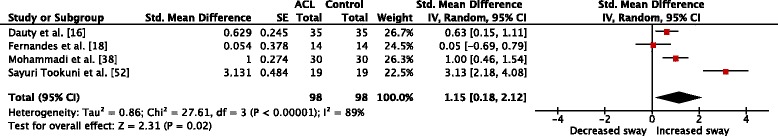
Forest plot comparison of mediolateral sway magnitude between the ACL-injured and ACL-matched control leg. *Std*. standardized, *SE* standard error, *IV* inverse variance, *CI* confidence interval, *df* degrees of freedom, *ACL* anterior cruciate ligament

**Fig. 5:**
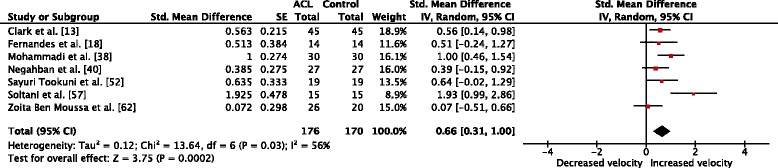
Forest plot comparison of sway velocity between the ACL-injured and ACL-matched control leg. *Std*. standardized, *SE* standard error, *IV* inverse variance, *CI* confidence interval, *df* degrees of freedom, *ACL* anterior cruciate ligament

Eight investigations in ACL patients [13, 16, 18, 27, 33, 52, 53, 57] compared sway magnitude of the injured leg to the matched leg of healthy controls, indicating a significantly increased total sway magnitude in the ACL group (Fig. 2, SMD_wm_ = 0.94, *p* = 0.003, CI = 0.32, 1.56, *I* ^2^ = 88%). A subsequent analysis also revealed large effects for increased sway magnitude in the anteroposterior direction (Fig. 3, SMD_wm_ = 0.58, *p* = 0.02, CI = 0.10, 1.06, *I* ^2^ = 62%) as well as the mediolateral direction (Fig. 4, SMD_wm_ = 1.15, *p* = 0.02, CI = 0.18, 2.12, *I* ^2^ = 89%) for the injured knee [16, 18, 38, 52]. Comparisons of sway velocity in the injured and matched limb were conducted in seven studies [13, 18, 38, 40, 52, 57, 62]. Collectively, the meta-analysis yielded a medium effect for sway velocity (SMD_wm_ = 0.66, *p* = 0.0002, CI = 0.31, 1.00, *I* ^2^ = 56%), indicating a significant increase in the ACL-injured leg compared to the control group (Fig. 5).

**Fig. 6:**
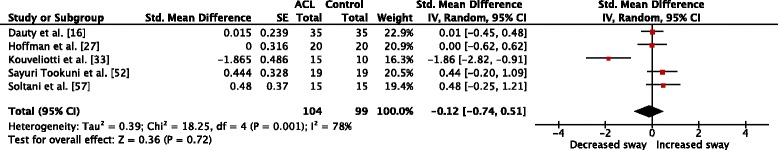
Forest plot comparison of sway magnitude between the ACL-non-injured and ACL-matched control leg. *Std*. standardized, *SE* standard error, *IV* inverse variance, *CI* confidence interval, *df* degrees of freedom, *ACL* anterior cruciate ligament

**Fig. 7:**
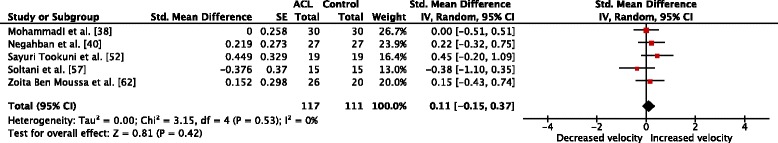
Forest plot comparison of sway velocity between the ACL-non-injured leg and ACL-matched control leg. *Std*. standardized, *SE* standard error, *IV* inverse variance, *CI* confidence interval, *df* degrees of freedom, *ACL* anterior cruciate ligament

Four trials assessed total sway magnitude [16, 27, 33, 52, 57] and sway velocity [38, 40, 52, 57, 62] for the ACL-non-injured leg. Among these studies, no significant difference has been found for comparisons of the non-injured and controls for neither total sway magnitude (Fig. 6, SMD_wm_ = −0.12, *p* = 0.72, CI = −0.74, 0.51, *I* ^2^ = 78%) nor sway velocity (Fig. 7, SMD_wm_ = 0.11, *p* = 0.42, CI = −0.15, 0.37, *I* ^2^ < 0.001%).

**Fig. 8:**
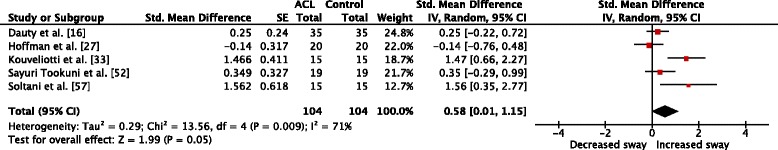
Forest plot comparison of sway magnitude between the ACL-injured leg and ACL-non-injured leg. *Std*. standardized, *SE* standard error, *IV* inverse variance, *CI* confidence interval, *df* degrees of freedom, *ACL* anterior cruciate ligament

**Fig. 9:**
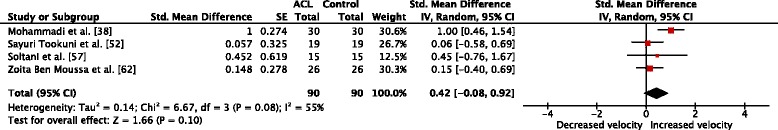
Forest plot comparison of sway velocity between the ACL-injured leg and ACL-non-injured leg. *Std*. standardized, *SE* standard error, *IV* inverse variance, *CI* confidence interval, *df* degrees of freedom, *ACL* anterior cruciate ligament

Statistically increased sway magnitude (Fig. 8, SMD_wm_ = 0.58, *p* = 0.05, CI = 0.01, 1.15, *I* ^2^ = 71%) was found for the comparison between the ACL-injured and ACL-non-injured leg [16, 27, 33, 52, 57], but no difference (Fig. 9, SMD_wm_ = 0.42, *p* = 0.10, CI = −0.08, 0.92, *I* ^2^ = 55%) was detected for sway velocity [38, 52, 57, 62].

## Discussion

The objective of this meta-analysis was to quantify postural stability during single-leg stance in patients after ACL injury compared to healthy controls. The comprehensive analysis revealed that postural stability was decreased in patients after ACL injury. During eyes open single-leg stance, patients showed significantly increased sway magnitudes and velocities in the injured limb. Additionally, postural sway was significantly increased in AP and ML direction. However, the non-injured side demonstrated no differences in sway magnitude or velocity compared to matched controls. Similarly, no difference in sway velocity has been observed among ACL patients between the injured and non-injured side, but sway magnitude significantly differed.

Following the model of Kapreli and Athanasopoulos [32], mechanoreceptor damage may lead to a disturbance of sensory transmission, contributing to alterations of afferent feedback and stabilizing reflexes that may implicate increased body sway [1, 8, 25, 59]. In line with Howells et al. [28] and Negahban et al. [41], the present findings support that postural sway is altered in patients after ACL injury. Medium to large effects were found for increased total sway magnitudes [13, 16, 18, 52, 53, 57], as well as increased anteroposterior and mediolateral sway in the injured leg [16, 18, 38, 52]. On the other hand, two studies [27, 33] found decreased postural sway in ACL patients compared to healthy controls. Differences in post-injury and post-surgical rehabilitation may explain the inconsistency of these results. While rehabilitation protocols commonly include balance training to positively influence clinical status and postural stability of ACL-injured patients [61], healthy controls may not be trained comparably for specific balance tasks and finally achieving worse results. Furthermore, the increases in AP and ML direction are consistent with previous reports [34, 37, 49, 59], also demonstrating postural impairments along these two axes. Since postural adjustments are limited in the knee joint, ankle and hip strategies may compensate for modified conditions to control the center of mass in AP and ML direction in relationship to the base of support [46, 50, 54]. Although compensational motor strategies may take part, ACL patients exhibit deficits in postural stability, supporting the supposition of a systematic change in sensorimotor control.

The present meta-analysis found no differences of postural sway between the non-injured and matched control leg. Previous systematic reviews [20, 41] have shown the non-injured leg to be affected by ACL injury. Similar to other reports [3, 42], one study [27] in this meta-analysis showed less postural sway in the non-injured limb compared to healthy controls. Nevertheless, other studies indicated a bilateral deficit of postural stability in ACL-injured patients [40, 52, 57]. Thus, higher-level sensorimotor control may be affected in addition to sensory afferent transmission. In fact, Baumeister et al. [7] found increased cortical processing in the brain related to ACL injury, also demonstrating significantly higher frontal brain activity in both the injured and non-injured leg.

However, in contradiction to earlier reports [22, 24, 42, 43], within-group differences of sway magnitudes were found between the ACL-injured and ACL-non-injured leg in the present meta-analysis. Future studies should apply neurophysiological measures to investigate the underlying mechanisms of sensorimotor processing after ACL injury.

Parameters of sway velocity were investigated in seven studies [13, 18, 38, 40, 52, 57, 62] revealing significant differences with medium to large effects for the comparison between the injured and matched leg. The mean sway velocity is arithmetically related to total path length of the CoP trajectory. It is usually calculated by total path length divided by trial duration [48, 49]. Thus, an increase in sway velocity may naturally be accompanied by increased sway magnitude, as demonstrated in this meta-analysis. With respect to the mathematical formula for mean sway velocity, the trial duration chosen for the assessment of postural stability may therefore crucially affect the outcomes. Moreover, other confounding variables may relate to differences in limb-matching procedures applied in the included studies. Howells et al. [28] suggested considering leg dominance as an influential factor for the comparison of ACL and healthy subjects. They found greater impairments in postural stability of the ACL group when compared to the dominant leg of healthy control. However, solely two of 11 included studies reported leg dominance. When evaluating a potential influence to the outcomes, future studies may explicitly provide detailed information about leg dominance.

### Limitations

Some limitations associated with the current review need to be considered. A major limitation was the heterogeneity of variance between studies. Except of one (Fig. 7), all other comparisons exceeded the recommended level of 50% heterogeneity [26]. Although studies were selected for highly specific criteria, the included research still exhibited variability. First, there are restraining factors inherent to the subject populations. One aim of this meta-analysis was to examine CoP measures in order to distinguish between a general population of ACL-injured patients and healthy individuals. Nevertheless, group differences may have affected the results of this systematic review. Further, influencing group factors may relate to sex distribution [29], age [35], or physical activity [61] in the experimental and control groups. Second, time frames for time after injury may influence the outcomes of ACL rehabilitation [34]. However, there is no evidence classifying the length of time after ACL injury and the related effects on postural stability. Future studies should investigate the time frames of ACL rehabilitation in more depth. Third, the research protocols considered in this systematic review differed in terms of methodological properties. In fact, none of the included examinations matched recent recommendations of Salavati et al. [51], to conduct five trials with duration of 60 s in assessments of postural control. Future studies may contemplate these methodological suggestions ensuring recordings to be mostly valid and consistent.

Fourth, matching of the injured to a matched limb of the control group was not consistently implemented throughout investigations. Since leg dominance was deemed to influence postural stability in ACL patients [11], conflicting methods for matching may distort the results of this meta-analysis. Information about the ratio of injuries to the dominant or non-dominant leg in ACL patients was solely provided in two studies [18, 38]. One study reported the injured leg being matched with the dominant leg of the control group [27]. When considering that leg dominance may differ between matched samples, divergent conclusions may result for postural stability. Collectively, the abovementioned methodological confounders may compromise the consistency of the findings and may therefore have contributed to the high levels of heterogeneity.

## Conclusions

The present meta-analysis indicates that postural stability in a standardized single-leg stance is impaired in patients after ACL injury. Furthermore, CoP measures appear to be suitable to differentiate ACL patients and healthy controls with respect to postural stability. Thus, the proposed measurement procedure may help physicians and physiotherapists to identify patients at greater risk for suffering a subsequent ACL injury and consequently allow adjusting their treatment or return to play strategies. Nevertheless, caution should be exercised when using the non-injured leg as a reference measure. However, the potential of these measures to provide further insights into underlying mechanisms of altered postural control is limited to theoretical considerations. While current investigations mainly describe motor responses to multimodal sensory feedback, further etiological approaches may assess neurophysiological mechanisms underlying functional deficits in ACL patients, providing valuable indications for diagnostics, rehabilitative treatment, or return to play assessment.
